# Predicting disease course in ulcerative colitis using stool proteins identified through an aptamer-based screen

**DOI:** 10.1038/s41467-021-24235-0

**Published:** 2021-06-28

**Authors:** Sanam Soomro, Suresh Venkateswaran, Kamala Vanarsa, Marwa Kharboutli, Malavika Nidhi, Ramya Susarla, Ting Zhang, Prashanth Sasidharan, Kyung Hyun Lee, Joel Rosh, James Markowitz, Claudia Pedroza, Lee A. Denson, Jeffrey Hyams, Subra Kugathasan, Chandra Mohan

**Affiliations:** 1 Department Biomedical Engineering, University of Houston, Houston, TX USA; 2 Department of Pediatrics, Emory University School of Medicine and Children Health Care of Atlanta, Atlanta, GA USA; 3 Center for Clinical Research and Evidence-based Medicine, McGovern Medical School, UT Health Science Center at Houston, Houston, TX USA; 4 Division of Gastroenterology, Hepatology, and Nutrition, Goryeb Children’s Hospital, Atlantic Health, Morristown, NJ USA; 5 Division of Gastroenterology, Hepatology, and Nutrition, Cohen Children’s Medical Center Of New York, New Hyde Park, NY USA; 6 Cincinnati Children’s Hospital Medical Center and the University of Cincinnati College of Medicine, Cincinnati, OH USA; 7 Division of Digestive Diseases, Hepatology, and Nutrition, Connecticut Children’s Medical Center, Hartford, CT USA; 8 Department of Human Genetics, Emory University School of Medicine, Atlanta, GA USA

**Keywords:** Predictive markers, Gastrointestinal diseases

## Abstract

In the search for improved stool biomarkers for inflammatory bowel disease (IBD), an aptamer-based screen of 1129 stool proteins was conducted using stool samples from an IBD cohort. Here we report that of the 20 proteins subsequently validated by ELISA, stool Ferritin, Fibrinogen, Haptoglobin, Hemoglobin, Lipocalin-2, MMP-12, MMP-9, Myeloperoxidase, PGRP-S, Properdin, Resistin, Serpin A4, and TIMP-1 are significantly elevated in both ulcerative colitis (UC) and Crohn’s disease (CD) compared to controls. When tested in a longitudinal cohort of 50 UC patients at 4 time-points, fecal Fibrinogen, MMP-8, PGRP-S, and TIMP-2 show the strongest positive correlation with concurrent PUCAI and PGA scores and are superior to fecal calprotectin. Unlike fecal calprotectin, baseline stool Fibrinogen, MMP-12, PGRP-S, TIMP-1, and TIMP-2 can predict clinical remission at Week-4. Here we show that stool proteins identified using the comprehensive aptamer-based screen are superior to fecal calprotectin alone in disease monitoring and prediction in IBD.

## Abstract (web summary)

Stool biomarkers hold promise for monitoring disease activity and predicting clinical course in inflammatory bowel disease (IBD) as they originate from the inflamed tissue. Here the authors report an aptamer-based proteomic screen, and discover several stool proteins that predict remission at four weeks.

## Introduction

The attributable risk of developing Inflammatory bowel disease (IBD) is about 0.5% in the general population, with IBD affecting ~1.6 million Americans, including as many as 80,000 children^1^. With over 20% of cases being diagnosed before the age of 17 IBD is one of the most common gastrointestinal chronic diseases affecting children and adolescents^2^. Since IBD is a lifelong disease, often treated with intense immunosuppressive therapies, a firm diagnosis supported by endoscopically obtained tissue biopsies, and histology is necessary for diagnosis. Since endoscopy is invasive and performed under general anesthesia in children, there is a need for noninvasive markers of clinical activity. Serological blood testing may aid in the diagnosis of IBD with current testing focusing on the detection of antimicrobial antibodies, but they are nonspecific and will not help with disease monitoring^3^. Serum C-reactive protein (CRP) is also often useful in distinguishing IBD from noninflammatory GI diseases such as irritable bowel syndrome but CRP is not specific to IBD inflammation^4^. Although serological testing and emerging serum biomarkers appear promising in IBD stratification, stool biomarkers hold great promise as a noninvasive test, as the stool is closer to the site of pathology and inflammation in IBD, and stool testing can be repeated as often as needed.

This study utilizes a high-throughput aptamer-based targeted proteomic assay to uncover stool biomarkers for pediatric IBD. With high dynamic range, sensitivity (fM to uM range), accuracy, and reproducibility^4,5^, this targeted screening platform that interrogates >1000 proteins, has been applied to several other diseases^6–16^. Candidate biomarkers discovered using this screening platform were validated by ELISA in cross-sectional and longitudinal cohorts of subjects. We take the opportunity to leverage the PROTECT cohort, a prospective pediatric UC inception cohort where treatment naïve baseline stool samples were collected, as well as three additional follow-up stool samples, to assess the clinical utility of these proteomic stool biomarkers in predicting clinical course in pediatric UC.

In this work, we demonstrate that the utility of comprehensive aptamer-based proteomic screens in identifying disease biomarkers for IBD that outperform the current gold standard, fecal calprotectin. The current study represents the first use of this aptamer-based screen in stool samples, and in IBD, representing the largest ever targeted stool proteomic study in IBD.

## Results

### Screening of pediatric IBD stool using an aptamer-based targeted proteomic assay

**Fig. 1:**
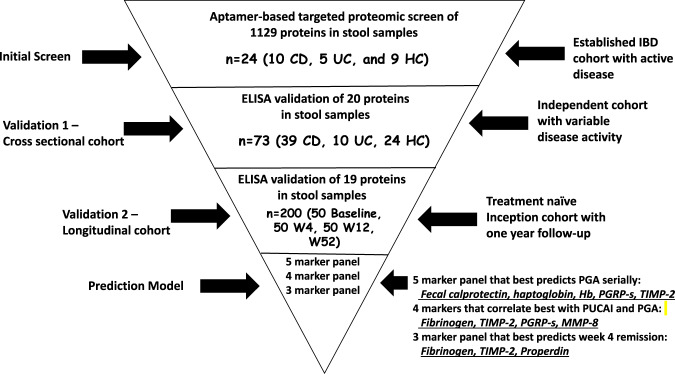
An overview of the study showing the discovery of panels of stool proteins arising from an initial aptamer-based screen. This consort diagram shows how the initial screen consisting of 1129 proteins were narrowed down to 19 stool proteins during validation. Various prediction models identified 7 stool proteins that could track clinical outcomes in a prospective pediatric UC cohort. Whereas the 4 markers, stool Fibrinogen, TIMP-2, PGRP-s, and MMP-8 correlate best with concurrent PUCAI and PGA scores, a 5-marker panel (Fecal calprotectin, haptoglobin, Hb, PGRP-s, and TIMP-2) best predict PGA longitudinally, while a 3-marker panel (Fibrinogen, TIMP-2, and Properdin) at baseline best predict week 4 remission. CD-Crohn’s disease, UC-ulcerative colitis, HC-healthy control, W4-week 4, W12-week 12, and W52-week52 of longitudinal follow-up.

**Fig. 2:**
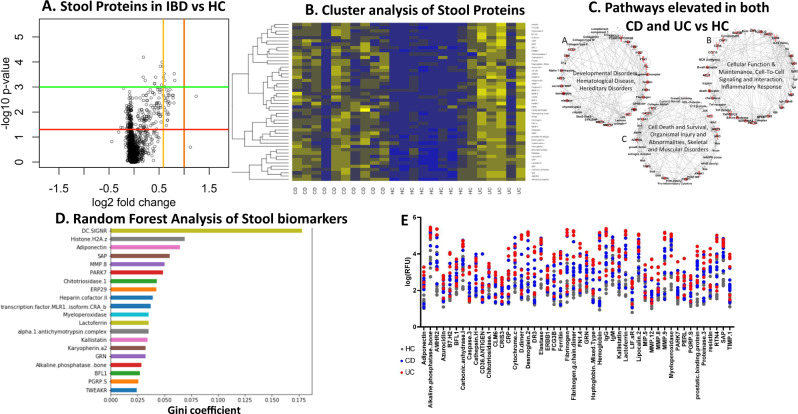
Aptamer-based screening of pediatric IBD stool samples for 1129 proteins. **A** A volcano plot representation of results of the aptamer-based screening of 1129 proteins analyzed in 24 pediatric stool samples (10 CD, 5 UC, and 9 healthy controls). Data were log-transformed and analyzed using a two-sided Mann–Whitney U-test to generate statistical *p*-values and *q*-values. 48 proteins were found to be elevated (*p* < 0.05, fold change > 1.25) in both CD and UC stool when compared to healthy control stool. Each dot represents one of the 1129 proteins and the *x*-axis shows the log_2_ transform of the fold change whereas the *y*-axis shows the −log_10_ transform of the *p*-value. Thresholds for fold change are indicated with yellow and orange vertical lines for fold change > 1.5 and fold change > 2 respectively while thresholds for *p*-value are indicated with horizontal red and green lines for *p* < 0.05 and *p* < 0.01 respectively. **B** A heatmap representation of the results of the aptamer-based screen showing the 48 proteins (*p* < 0.05, fold change > 1.25) elevated in IBD stool. Proteins that are above the mean value (for each biomarker) are yellow, while those below the mean are blue. **C** Ingenuity pathway analysis depicts proteins that were significantly elevated in the stools of both CD and UC patients when compared to healthy control stool, and the functional pathways they belong to molecules elevated in IBD stool when compared to healthy control stool are shaded red. Documented and putative interactions between the displayed molecules are indicated by solid and dashed arrows, respectively. **D** Random forest classification analysis identification of the 20 most discriminatory stool proteins with the largest impact on distinguishing IBD subjects from healthy controls, ordered by their GINI coefficient. **E** The top 48 proteins (ranked in order of fold change for IBD versus healthy controls) that were significantly elevated in both CD and UC stool when compared to healthy control stool are shown as a dot plot where CD, UC, and healthy control subjects are shown using blue, red, and gray dots, respectively. Of note, 3 of these 48 stool proteins also survived multiple testing corrections, with *q*-values < 0.05.

An overview of the study flow is depicted in Fig. 1. For the initial aptamer-based screen of stool proteins, 24 stool samples were interrogated for 1129 proteins, as detailed in the methods section. Of the 1129 proteins assayed using the aptamer-based screen, significant upregulation of multiple proteins was seen in IBD stool compared to healthy controls, as shown in the volcano plot (Fig. 2a). Of the proteins that were significantly elevated in IBD vs HC, 48 stool proteins were found to be elevated (*p* < 0.05 and fold change > 1.25) in both CD and UC stool when compared to healthy control stool (Fig. 2b). Of these 48 stool proteins, only 3 survived multiple testing corrections (*q* < 0.05), presumably because of the small sample size. Two proteins were elevated in the stool of CD patients but not UC when compared to healthy controls, while 18 proteins were elevated in the stool of UC patients when compared to healthy controls while not being elevated in CD stools compared to the healthy controls (Supplementary Fig. 1).

The proteins that were significantly elevated in the stool of both CD and UC clustered into several inter-related functional networks by pathway analysis, including (1) developmental disorders, hematological diseases, hereditary disorders, (2) cellular function and maintenance, cell-to-cell signaling and interaction, inflammatory response, and (3) cell death and survival, organismal injury, as shown in Fig. 2c, with the proteins elevated in the diseased stools being displayed in red. In addition, Random Forest Analysis also implicated DC-SIGNR, adiponectin, GRN, and MMP-12 as additional discriminatory molecules with the largest impact on IBD versus healthy control discrimination (Fig. 2d). Based on the aptamer-based screen, 33 proteins were selected for ELISA validation; the selected proteins and the reasons for selecting them are listed in Supplementary Table 3. Calprotectin and Lysozyme were also assayed as a “gold-standard” for comparison, as these stool proteins have been well documented to be elevated in the IBD literature.

### Validation of stool protein biomarkers in pediatric IBD stool by ELISA

**Fig. 3:**
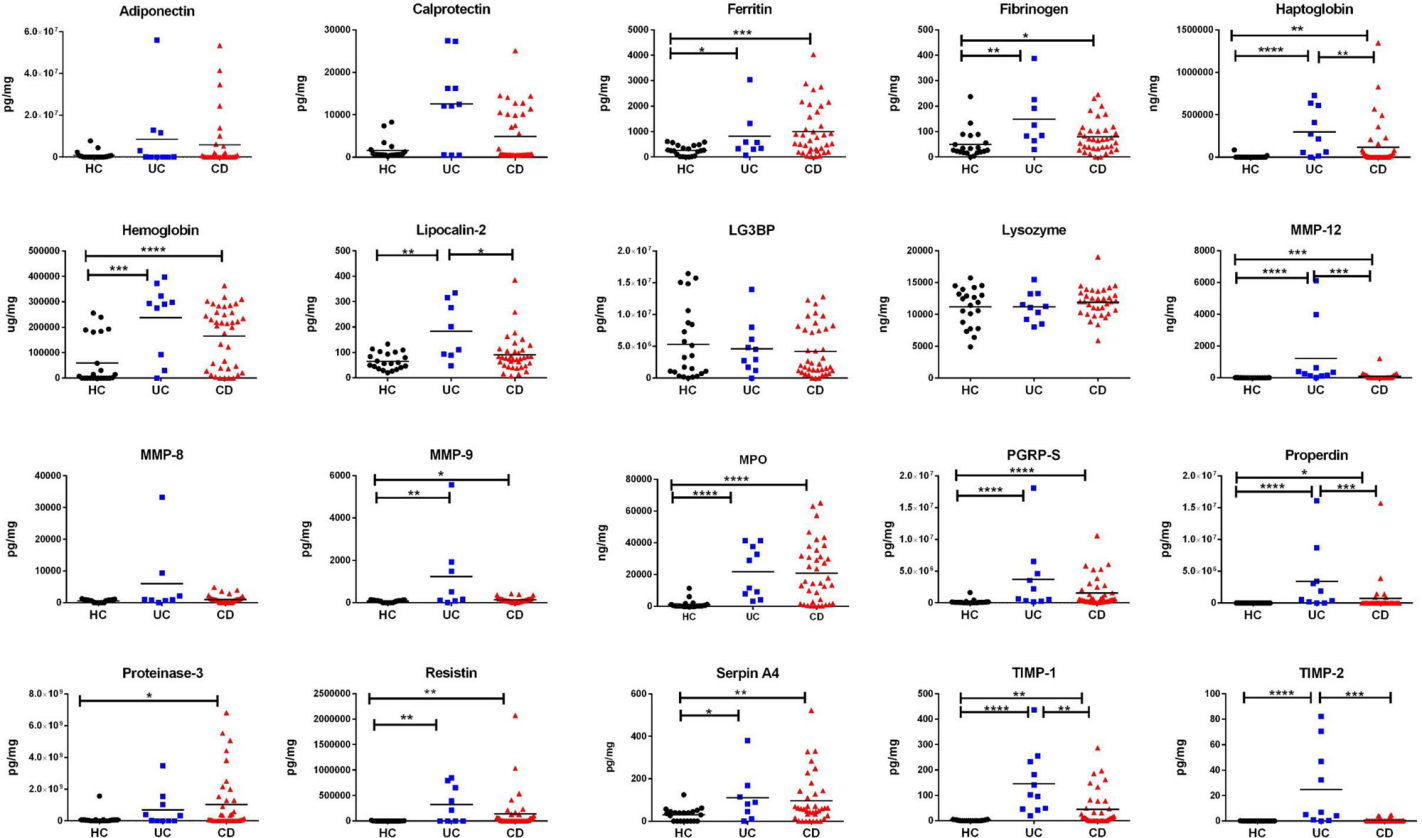
Cross-sectional ELISA validation of elevated stool proteins in pediatric IBD stool. Shown are the ELISA validation results of 20 stool proteins in a cross-sectional cohort of 73 pediatric subjects (24 healthy controls, 10 UC, and 39 CD). The concentration of each protein was assayed in the 73 subjects’ stool and then normalized to mg of a stool sample. The horizontal line represents the mean of each subject group. *p < 0.05, **p < 0.01, ***p < 0.001, and ****p < 0.0001 as determined using two-sided Mann–Whitney U-test.

Of the 33 molecules initially selected for ELISA validation, only 20 could be detected in stool samples at a sample dilution of at least 1:2. These were next assayed by ELISA in a cohort of 73 stool samples, drawn from 39 CD patients, 10 UC patients, and 24 healthy controls, and normalized by stool weight. As can be seen in Fig. 3 and Table 1, Stool Ferritin, Fibrinogen, Haptoglobin, Hemoglobin, Lipocalin-2, MMP-12, MMP-9, Myeloperoxidase, PGRP-S, Properdin, Resistin, Serpin A4, and TIMP-1 are all significantly elevated (*p* < 0.05) in both UC and CD stool compared to healthy controls. Calprotectin, Proteinase-3, and TIMP-2 are significantly elevated in CD stool versus healthy control but not significantly elevated in the stool of UC subjects. Receiver operating curve analysis ordered the stool markers that best distinguish UC from healthy controls as follows: TIMP-1 (AUC = 1.00), MMP-12 (AUC = 0.98), MPO (AUC = 0.97), PGRP-S (AUC = 0.96), TIMP-2 (AUC = 0.95), Haptoglobin (AUC = 0.91), Properdin (AUC = 0.89), and Hemoglobin (AUC = 0.87), in order of decreasing AUC value, as shown in Table 1.

**Table 1:** Stool protein markers that best distinguish UC from HC, or CD from HC, based on ELISA validation assays.

Protein^1^	Healthy	U. Colitis	Crohn’s	Ulcerative colitis versus healthy control					Crohn’s disease versus healthy control				
	Mean (median)	Mean (median)	Mean (median)	Fold Change^2^	Cut-off^3^	AUC	Sensitivity	Specificity	Fold change^2^	Cut-off^3^	AUC	Sensitivity	Specificity
**Adiponectin**^**n**^	738 (19)	8403 (102)	5088 (55)	11.4	3146	0.62(0.38–0.84)	0.40(0.12–0.74)	0.92(0.73–0.99)	6.9	871	0.57(0.43–0.70)	0.33(0.19–0.50)	0.88(0.67–0.97)
**Calprotectin**^**p**^	2355 (651)	12540 (12,261)	5036 (672)	5.3	12118	0.7(0.47–0.98)	0.70(0.34–0.93)	0.96(0.79–0.99)	2.1***	9898	0.59(0.44–0.73)	0.31(0.17–0.47)	0.96(0.78–0.99)
**Ferritin**^**n**^	291 (245)	658 (335)	951 (532)	2.3*	312	0.60(0.36–0.85)	0.70(0.35–0.93)	0.63(0.41–0.81)	3.3***	646	0.72(0.59–0.84)***	0.49(0.32–0.65)	0.96(0.78–0.99)
**Fibrinogen**^**n**^	131 (30)	119 (84)	75 (65)	0.9**	64	0.62(0.38–0.85)	0.70(0.34–0.93)	0.71(0.49–0.87)	0.6*	38	0.60(0.45–0.76)	0.69(0.52–0.83)	0.63(0.40–0.81)
**LG3BP**^**n**^	5120 (2490)	4601 (3722)	4204 (2238)	0.9	1225	0.53(0.32–0.73)	0.90(0.56–0.99)	0.42(0.22–0.63)	0.8	1155	0.51(0.35–0.66)	0.77(0.60–0.88)	0.42(0.22–0.63)
**Haptoglobin**^**µ**^	5 (0)	300 (246)	119 (6)	61.4****	13	0.91(0.77–1.05)****	0.90(0.55–0.99)	0.92(0.73–0.99)	24.3**	3	0.71(0.59–0.83)**	0.56(0.39–0.72)	0.92(0.73–0.99)
**Hemoglobin**^**m**^	57 (1)	237 (292)	166 (214)	4.2***	276	0.87(0.70–1.02)****	0.70(0.35–0.93)	1.00(0.85–1.00)	2.9****	21	0.79(0.68–0.91)****	0.82(0.66–0.92)	0.67(0.44–0.84)
**Lipocalin-2**^**n**^	58 (53)	147 (102)	87 (75)	2.5**	88	0.70(0.45–o.094)	0.70(0.35–0.93)	0.75(0.53–0.90)	1.5*	63	0.63(0.49–0.77)	0.67(0.49–0.81)	0.63(0.41–0.81)
**Lysozyme**^**µ**^	10 (11)	11 (11)	10 (12)	1.1	8	0.55(0.35–0.76)	1.00(0.85–1.00)	0.33(0.16–0.55)	1.1	9	0.52(0.36–0.68)	0.82(0.66–0.92)	0.38(0.18–0.59)
**MMP-12**^**n**^	2 (0)	1215 (301)	85 (10)	763.4****	111	0.98(0.93–1.02)****	0.90(0.55–0.99)	1.00(0.85–1.00)	53.4***	18	0.74(0.64–0.85)****	0.44(0.28–0.60)	1.00(0.85–1.00)
**MMP-8**^**n**^	539 (684)	4821 (886)	991 (822)	8.9	1048	0.62(0.39–0.86)	0.40(0.12–0.74)	0.92(0.73–0.99)	1.8	1003	0.62(0.48–0.75)	0.44(0.28–0.60)	0.92(0.73–0.99)
**MMP-9**^**n**^	59 (68)	984 (140)	129 (99)	16.7**	161	0.70(0.47–0.93)	0.50(0.12–0.81)	1.00(0.85–1.00)	2.2*	162	0.68(0.55–0.82)**	0.36(0.21–0.52)	1.00(0.85–1.00)
**Myeloperoxidase**^**n**^	1264 (361)	21846 (20,293)	21657 (15675)	17.3****	3301	0.97(0.93–1.01)****	1.00(0.85–1.00)	0.91(0.72–0.99)	17.1****	2739	0.90(0.84–0.97)****	0.74(0.58–0.87)	0.91(0.72–0.99)
**PGRP-S**^**n**^	143 (84)	3687 (1417)	1537 (513)	25.8****	172	0.96(0.91–1.01)****	1.00(0.85–1.00)	0.88(0.67–0.97)	10.7****	126	0.88(0.80–0.97)****	0.87(0.73–0.96)	0.83(0.62–0.95)
**Properdin**^**n**^	0 (0)	3422 (1212)	625 (0)	3421629.6****	193	0.89(0.74–1.03)****	0.80(0.44–0.97)	1.00(0.85–1.00)	625347.4*	44	0.55(0.46–0.64)	0.21(0.09–0.36)	1.00(0.85–1.00)
**Proteinase-3**^**µ**^	92 (30)	680 (177)	1024 (83)	7.4	336	0.68(0.46–0.90)	0.50(0.19–0.81)	0.96(0.78–0.99)	11.1*	73	0.69(0.55–0.82)**	0.54(0.37–0.69)	0.96(0.79–0.99)
**Resistin**^**n**^	2 (0)	290 (108)	139 (6)	162.4**	212	0.75(0.55–0.96)*	0.50(0.18–0.81)	1.00(0.85–1.00)	77.9**	6	0.72(0.60–0.84)**	0.51(0.35–0.68)	0.96(0.79–0.99)
**Serpin A4**^**n**^	26 (28)	98 (81)	92 (57)	3.7*	81	0.73(0.50–0.96)*	0.56(0.21–0.86)	0.96(0.79–0.99)	3.5**	57	0.72(0.59–0.84)***	0.51(0.35–0.67)	0.92(0.73–0.99)
**TIMP-1**^**p**^	1 (0)	156 (122)	42 (11)	145.6****	20	1.00(1.00–1.00)****	1.00(0.85–1.00)	1.00(0.85–1.00)	39.3**	9	0.74(0.63–0.85)****	0.54(0.37–0.69)	1.00(0.85–1.00)
**TIMP-2**^**p**^	0 (0)	25 (6)	0 (0)	3494.2****	0.44	0.95(0.85–1.05)****	0.90(0.55–0.99)	1.00(0.85–1.00)	66.6	0.37	0.58(0.51–0.66)*	0.21(0.09–0.36)	1.00(0.85–1.00)

^1^Biomarker proteins are listed bolded for clarity; n = ng/ml, p = pg/ml, µ = ug/ml, m = mg/ml.

^2^Fold change reflects disease vs HC. Shown *p*-values are determined using the Mann–Whitney U test (*P < 0.05, **P < 0.01, ***P < 0.001, ****P < 0.0001).

^3^Cut-off values for AUC were determined using the Youden index.

Similarly, the markers that best distinguish CD from healthy controls are MPO (AUC = 0.91), PGRP-S (AUC = 0.89), Hemoglobin (AUC = 0.80), MMP-12 (AUC = 0.74), TIMP-1 (AUC = 0.74), Resistin (AUC = 0.73), Serpin A4 (AUC = 0.72), Ferritin (AUC = 0.72), Haptoglobin (AUC = 0.71), MMP-9 (AUC = 0.69), Proteinase-3 (AUC = 0.69), and TIMP-2 (AUC = 0.59), in order of decreasing AUC value. Of these, stool Ferritin, MMP-9, and Proteinase-3 are only able to distinguish CD but not UC from healthy controls (Table 1). In terms of specificity, several stool proteins exhibited very high specificity (0.9–1.0) for both UC and CD, including MMP-12, MMP-8, Properdin, Resistin, TIMP-1, and TIMP-2 (Table 1). Whereas several stool proteins exhibited very high sensitivity (1.00) for detection of UC (Lysozyme, MPO, PGRP-S, and TIMP-1), stool PGRP-S exhibits the highest sensitivity for detecting CD (sensitivity = 0.87), as tabulated in Table 1.

Although several of the proteins listed above exhibited promising associations with disease severity and/or remission, firm conclusions could not be drawn owing to the limited sample size. However, we have performed such an analysis in the longitudinal PROTECT cohort, as detailed below.

### Longitudinal evaluation of stools proteins using the PROTECT cohort

**Fig. 4:**
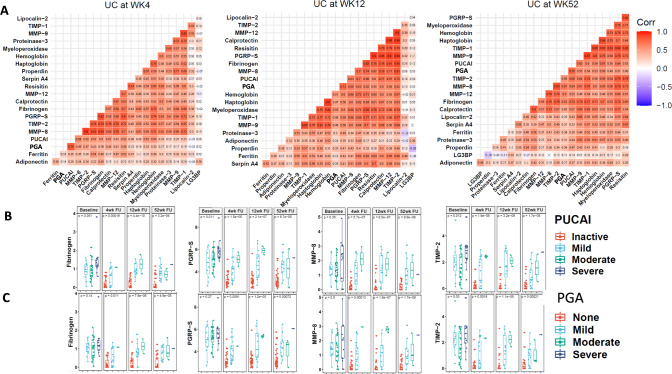
Longitudinal analysis of stool protein biomarkers in PROTECT Study participants. **A** A pairwise Pearson’s correlation analysis was performed for 19 stool proteins along with PUCAI and PGA disease severity indices across three follow-up time points, WK4, WK12, and WK52, in 50 UC patients from the PROTECT cohort. At each time point, the markers that are strongly correlated with PUCAI and PGA were grouped based on their correlation coefficient. Pearson correlations that are not significant at *p* < 0.05 are crossed out. As a result, 4 markers “Fibrinogen”, “MMP-8”, “PGRP-S”, and “TIMP-2” were identified since they show a strong positive correlation with PUCAI and PGA disease severity index scores at most of the follow-up time-points. **B** Boxplot representation shows the association of 4 selected stool proteins with the PUCAI disease severity groups (Inactive, Mild, Moderate, and Severe) across multiple time-points, in 50 UC patients from the PROTECT cohort. Shown p-values were calculated using the *anova* test in R. **C** Boxplot representation showing the associations of each selected stool protein with PGA disease severity groups, (None, Mild, Moderate, and Severe) across multiple time-points. Shown *p*-values were calculated using the *anova* test in R. In each boxplot the middle line represents the mean value and outer two-line bars represent the data range. Symbols shown outside the bar range indicate outliers.

Next, these proteins were evaluated in a longitudinal cohort of 50 pediatric IBD patients at 4 time-points from the PROTECT study (Supplementary Table 2). In total 19 out of the 20 proteins that were validated by ELISA in the cross-sectional cohort (Table 1) were successfully evaluated in this longitudinal cohort, while Lysozyme was omitted as the assay failed to meet quality control criteria. At all time-points, PUCAI and PGA scores were correlated well with each other, as expected (Fig. 4). Of the 19 stool proteins tested, 4 stool proteins namely Fibrinogen, MMP-8, PGRP-S, and TIMP-2 show the strongest positive correlation with PUCAI and PGA scores at most of the time-points, with correlation coefficients ranging from 0.5–0.72, being higher than the correlation coefficient exhibited by fecal calprotectin.

**Fig. 5:**
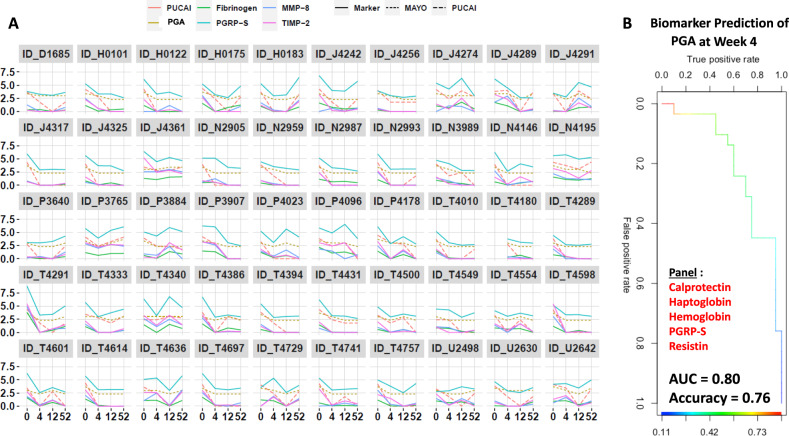
Fecal Fibrinogen, MMP-8, PGRP-S, and TIMP-2 track with PUCAI and PGA indices in 50 UC patients from the PROTECT cohort followed up at 4 serial visits. **A** Expression of 4 selected stool proteins along with PUCAI and PGA disease severity index scores are plotted for each patient (total *N* = 50 UC patients from the PROTECT cohort). The *X*-axis shows the four-time points, whereas *Y*-axis shows the log-transformed values of stool makers, PUCAI, and PGA disease severity scores. **B** Biomarker panels that best predict concurrent disease activity (PGA Index at Week 4 follow up) were determined using elastic-net regularized logistic regression, after adjusting for age, gender, ethnicity, and medication use. The best panel comprises stool Calprotectin, Haptoglobin, Hemoglobin, PGRP-S, and Resistin, with an accuracy of 0.76 and AUC of 0.80 (95% CI: 0.68–0.93). Indeed, this prediction accuracy increases to 0.80, when the model is further adjusted for the PGA score at baseline.

Additive analysis by ANOVA show that the selected stool proteins were able to distinguish UC disease severity groups at WK4, at WK12 and at WK52. We observed the same trend for both PUCAI and PGA disease severity indexes (Fig. 4b, c). As shown by Fig. 4b, c, worsening disease severity is significantly associated with a progressive increase in stool Fibrinogen, MMP-8, PGRP-S, and TIMP-2, irrespective of whether PUCAI or the PGA index was used. We also examined the temporal expression profile of these four stool proteins along with their PUCAI and PGA scores in each patient individually, over four serial visits. Similarly, we also tested the associations of these selected proteins with the Endoscopic MAYO score at WK52 and observed a nominal significant association for all the makers (Supplementary Fig. 3). Almost all the patients show a similar pattern for all 4 stool markers and UC disease severity index scores, suggesting that these stool proteins faithfully track disease activity in UC (Fig. 5a).

After adjusting for age, gender, ethnicity, and medication use, a 5-marker panel comprised of stool Calprotectin, Haptoglobin, Hemoglobin, PGRP-S, and Resistin measured at W4 best predict the PGA score at W4, with an accuracy of 0.76 and AUC of 0.80 (95% CI: 0.68–0.93), as determined using elastic-net regularized logistic regression. The prediction accuracy increases to 0.80, when the model is further adjusted for the PGA score at baseline (Fig. 5b). Similarly, after adjusting for age, gender, ethnicity, and medication use, a 5-marker panel comprised of longitudinal values (entered as time-varying covariates) of stool Calprotectin, Haptoglobin, Hemoglobin, PGRP-S, and TIMP-2 best track with the PGA score over the 4 follow-up time-points, as determined using Bayesian generalized multilevel models with horseshoe prior (df = 3, par_ratio = 0.5) in a proportional odds logistic model. A similar analysis revealed that a panel comprised of stool Calprotectin, PGRP-S, Serpin A4, Adiponectin, and TIMP-2 (entered as time-varying covariates) best track with the PUCAI index over the 4 follow-up time-points with the same prior in a linear regression model with Bayesian ${R}^{2}$ 0.58 (the proportion of predicted variance explained by the model^17^).

### Baseline stool markers as predictors of clinical and treatment outcomes in UC

**Fig. 6:**
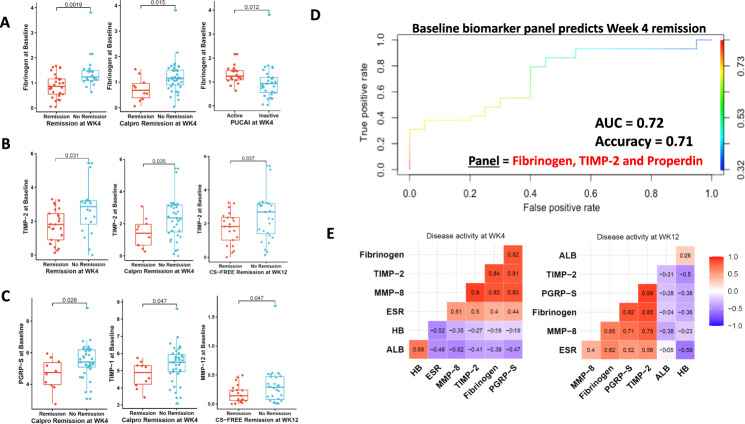
Selected baseline stool proteins predict clinical and treatment outcomes in UC. **A** Boxplots for stool Fibrinogen at baseline parsed by WK4 clinical outcome. WK4 Remission, WK4 Calprotectin-defined Remission, and WK4 PUCAI active disease show signfiicant association with fecal Fibrinogen at baseline (*p* < 0.05). **B** Boxplots for stool TIMP-2 at baseline parsed by WK4 Remission, WK4 Calprotectin-defined Remission, and WK12 CS-FREE Remission. **C** Boxplots for other significant associations. Shown are stool PGRP-S, TIMP-1, and MMP-12 proteins at baseline plotted against WK4 Calprotectin-defined Remission and WK12 CS-FREE Remission. In the boxplots, *Y*-axis shows log-transformed stool protein values at baseline and the *X*-axis shows the various clinical outcomes at subsequent follow-up time-points. In each boxplot, the middle line represents the mean value and the outer two-line bars represent the data range. The symbols outside the bar range indicate outliers. Shown *p*-values were calculated using two-sided Wilcoxon Test in R. **D** After adjusting for age, gender, ethnicity and medication use, a 3-marker panel comprised of stool Fibrinogen, TIMP-2, and Properdin measured at baseline best predict clinical remission at W4, with an accuracy of 0.71 (AUC = 0.72; 95% CI: 0.57–0.87), as determined using elastic-net regularized logistic regression. **E** A pairwise Pearson’s correlation was performed for the selected stool markers against conventional blood markers (Hemoglobin (HB), ESR (Erythrocyte sedimentation Rate), and Albumin (Alb) at WK4 or WK12. Correlations that are not significant (*p* > 0.05) are crossed out.

Among the four selected stool proteins, stool Fibrinogen at baseline is able to predict WK4 Remission, WK4 Calprotectin-defined Remission, and WK4 PUCAI (Fig. 6a), with these outcome measures being defined in Supplementary Table 2. Similarly, stool TIMP-2 at baseline is able to predict WK4 Remission, WK4 Calprotectin-defined Remission, and WK12 CS-FREE Remission (Fig. 6b). We also observed that stool PGRP-S, TIMP-1, and MMP-12 proteins at baseline are able to predict WK4 Calprotectin-defined Remission and WK12 CS-FREE Remission during follow-up (Fig. 6c). Compared to these stool proteins, stool calprotectin at baseline is not able to predict any of these clinical outcomes during follow-up (Supplementary Fig. 2).

After adjusting for age, gender, ethnicity, and medication use, a 3-marker panel comprised of stool Fibrinogen, TIMP-2, and Properdin measured at baseline best predict clinical remission at W4, with an accuracy of 0.71 and AUC of 0.72 (95% CI: 0.57–0.87), as determined using elastic-net regularized logistic regression (Fig. 6d). When we employed an upsampling strategy (due to potential problems that may arise from the class imbalance)^18,19^, Lasso penalized regression model identified a 5-marker panel, comprised of baseline stool PGRP-S, Myeloperoxidase, Resistin, TIMP2, and Acrp30 as being most predictive of clinical remission (“Remission_CAL”) at W4, with a very high accuracy 0.97.

Finally, all 4 stool proteins, Fibrinogen, TIMP-2, PGRP-S, and MMP-8 also correlate significantly with other laboratory markers of disease at W4 and/or at W12, including ESR, reduced albumin, and reduced hemoglobin (Fig. 6e).

### Week 4 stool markers as predictors of long-term outcomes in UC

We assessed whether early changes in stool biomarkers while on therapy (assayed at week 4) can predict outcomes further on (at week 12 and 52). We undertook three separate analyses: (1) for each outcome of PGA, PUCAI, and Remission_CAL (clinical remission with normal calprotectin) at Week 12 and Week 52 (separately), we used an elastic net model including all 19 biomarkers measured at Week 4 as predictors adjusting for age, gender, ethnicity, and medication use. From this model, we identified the best predictors of each outcome at each of the two-time points. (2) We used logistic or linear regression to predict each of the outcomes at Week 12 and Week 52 using the 3-marker panel described in Fig. 6 consisting of Fibrinogen, TIMP2 and Properdin measured at Week 4. (3) We used logistic or linear regression to predict each of the outcomes at Week 12 and Week 52 using the 5-marker panel described in Fig. 5 consisting of Calprotectin, Resistin, Haptoglobin, PGRP-S, and Hemoglobin measured at Week 4.

Results from these analyses are summarized in Table 2. Briefly, after adjusting for age, gender, ethnicity and medication use, a single marker panel comprised of LCN2 measured at W4 best predict the PGA score at W12, with an accuracy of 0.67 and AUC of 0.72, as determined using elastic-net regularized logistic regression. Both the 3-marker panel and 5-marker panels, measured at W4, perform equally well or better in predicting W12 PGA, with an accuracy of 0.78 and AUC of 0.80–0.81. Both these panels are also able to predict W52 PGA, unlike the Elastic Net model (Table 2).

**Table 2:** Comparison of AUC, Accuracy, and MSE2 values of stool protein biomarkers at week 4 in predicting long-term outcome.

^1^*“Remission”* refers to CAL_Remission, ^2^*MSE* mean Squared Error.

Whereas outcomes at W12 are shaded as gray rows, outcomes at W52 are shaded as white rows.

After adjusting for age, gender, ethnicity, and medication use, the panel comprised of MMP12, Haptoglobin, SerpinA4, Proteinase, and LCN2 best predict Remission_CAL at W52 with an accuracy of 0.90 and AUC of 0.94, as determined using elastic-net regularized logistic regression. Both the 3-marker and 5-marker panels, measured at W4, performed equally well in predicting W12 Remission_CAL as well as W52 Remission_CAL with reasonably strong accuracy and AUC values (Table 2). In contrast, all panels assayed at W4 perform poorly at predicting W12 and W52 PUCAI.

## Discussion

Research over the past several years has uncovered potentially important stool biomarkers for inflammatory bowel disease. Most importantly, calprotectin, a stool biomarker widely used clinically, is a protein released by damaged white blood cells (granulocytes, monocytes, and macrophages) and epithelial cells^20^. It serves as a marker of neutrophil migration within the GI tract and has a higher specificity than other inflammatory markers commonly used in clinical practice, such as C-reactive protein^21^. Levels of fecal calprotectin have been shown to correlate well with endoscopy and histopathologic metrics of disease activity and disease recurrence^22,23^. However, fecal calprotectin has several limitations. The sensitivity and specificity of fecal calprotectin testing are dependent on the location of the inflammation. Several studies reported lower specificity in CD patients versus UC patients, and higher specificity for large bowel disease versus small bowel disease^24^. Approximately 80% of CD patients have ileal involvement and up to 32% have isolated small bowel disease. This may explain the limited use of calprotectin for small bowel disease, hence its debatable use in the diagnosis of CD^25^. Sensitivity and specificity have been shown to increase with age. Hence, fecal calprotectin may have limitations in the diagnosis, monitoring of disease progression, and prediction of disease relapse in younger children^26^.

As of now, it is unknown if there could be other stool proteins that might be superior to fecal Calprotectin in their predictive performance in IBD, because a comprehensive unbiased screen of stool proteins has never been reported in IBD. The present work represents the first attempt through an aptamer-based search for additional stool protein biomarkers. This study has uncovered several stool proteins that outperform fecal calprotectin in many respects. In the cross-sectional IBD cohort (Table 1, Fig. 3), several stool proteins (Haptoglobin, MMP-12, MPO, PGRP-S, Properdin, TIMP-1) significantly discriminate UC from HC with AUCs from 0.89–1.00 (*p* < 0.0001), compared to Calprotectin (AUC = 0.73; not significant). Likewise, several stool proteins (Hemoglobin, MMP-12, MPO, PGRP-S, and TIMP-1) significantly discriminate CD from HC with AUCs from 0.74–0.91 (*p* < 0.0001), compared to Calprotectin (AUC = 0.59; not significant). In the longitudinal study of the PROTECT cohort, Calprotectin shows no significant correlation with PUCAI or PGA scores (Fig. 4), whereas stool Fibrinogen, MMP-8, PGRP-S, and TIMP-2 correlate with PUCAI and PGA scores at most of the time-points, with correlation coefficients ranging from 0.5 to 0.72. Furthermore, baseline calprotectin fails to predict WK4 Calprotectin-defined Remission or WK12 CS-FREE Remission (Supplementary Fig. 2), whereas baseline stool Fibrinogen, MMP-12, PGRP-S, TIMP-1, and TIMP-2 predict some aspect of remission at W4 and/or W12 (Fig. 6). A 3-marker panel comprised of stool Fibrinogen, TIMP-2 and Properdin measured at baseline predict Remission at W4 with a prediction accuracy of 0.71 and AUC of 0.72, compared to Calprotectin, whose prediction accuracy and AUC were 0.59 and 0.60, respectively. Thus, by various measures, several other stool proteins outperform fecal calprotectin as biomarkers for IBD. In view of their biomarker potential and functional properties, these stool proteins merit further investigation, including hemoglobin, MMP-8^27^, MMP-9^28^, MMP-12, MPO^29^, lipocalin-2^30^, PGRP-S^31^, TIMP-1^27,32^, TIMP-2^27,33^, and Adiponectin^34,35^.

Matrix metalloproteinases (MMP-8, MMP-9, and MMP-12) are a group of zinc-dependent proteolytic enzymes that play an important role in remodeling the extracellular matrix (ECM)^27^. Previous studies have demonstrated elevated MMP-8 in murine colitis and IBD^36,37^. In the present study, fecal MMP-8 levels are elevated in both CD and UC patients (Fig. 3). Data from the longitudinal UC study show a significant correlation between fecal MMP-8 and disease severity in UC, at weeks 4, 12, and 52, suggesting that this protein may be used to predict PUCAI and PGA disease severity (Fig. 4a, b). This correlation is further illustrated in Fig. 5. In addition, fecal MMP-8 shows a strong positive correlation with PUCAI and PGA scores, suggesting it may be clinically utilized for monitoring disease activity and outcomes, as well as a preendoscopic test. Fecal MMP-8 exhibits a stronger correlation coefficient with disease activity when compared to fecal calprotectin.

Tissue inhibitors of metalloproteinases (TIMP) are natural inhibitors of matrix metalloproteinases. An adequate balance of MMP and TIMP activity is essential for normal extracellular matrix remodeling and functioning. An imbalance of MMP and TIMP activity has been correlated to the active inflammation seen in IBD, with an increase in the activity of various MMP and TIMP molecules^38,39^. It has been reported that serum TIMP-2 may serve as an important biomarker of disease remission and treatment response^33^. The present study demonstrates that increased fecal TIMP-1 and TIMP-2 are able to distinguish UC from HC (AUC = 0.95), with high specificity (>90%). In the longitudinal study, fecal TIMP-2 strongly correlates with PUCAI and PGA scores, and baseline fecal TIMP-2 is one of the best predictors of Week 4 remission, Week 4 Calprotectin defined remission, and Week 12 CS-Free remission. Indeed, after correction for patient demographics and medication use, fecal TIMP-2 is the only protein that is included in the biomarker panel for predicting subsequent disease remission, and for longitudinal disease tracking, using PUCAI or the PGA index. Together with past reports^33^, the present finding offers resounding support for the use of fecal TIMP-2 for predicting treatment response and for tracking disease progression and remission serially.

Fibrinogen, a key player in blood coagulation and inflammation^40^, has been reported to be raised in UC serum^41^. In the present cross-sectional study, fecal fibrinogen is significantly elevated (Fig. 2b, e) in both UC vs HC and CD vs HC groups (*p* < 0.05), with high AUC values. Longitudinal data demonstrate a strong positive correlation between fecal fibrinogen and PUCAI scores. Fecal fibrinogen increases proportionately with disease severity in UC, irrespective of whether PUCAI or the PGA index is used to assess disease severity. Baseline fecal fibrinogen also shows significant ability to predict Week 4 remission and Wk4 Calprotectin-defined remission, alluding to its potential use as a predictor of disease recurrence. When the analysis is adjusted for patient demographics and medication use, fecal fibrinogen is only one of 3 proteins (besides TIMP-2 and properdin) included in the baseline multi-marker panel that best predict subsequent disease remission.

Peptidoglycan recognition proteins (PGRP) are a group of bacterial recognition proteins that function as part of the innate immune system that serves to maintain a normal gut microbiome. Altered circulating PGRP is associated with IBD^42^. Genetic polymorphisms in PGRP-S (PGLYRP1) have been strongly associated with UC^43^. In the present study, fecal PGRP-S levels are elevated in both CD and UC (*p* < 0.05) and show significant ability to differentiate UC from healthy controls (AUC = 0.96) and CD from healthy controls (AUC = 0.89). Perhaps most impressive is the observation that fecal PGRP-S demonstrates the highest sensitivity for detection of UC (100%) and CD (87%), making it an ideal biomarker for screening populations at risk. Moreover, baseline fecal PGRP-S is able to successfully predict Week 4 calprotectin-defined remission and Week 12 CS-Free remission. After correction for patient demographics and medication use, fecal PGRP-S qualifies for inclusion within the best biomarker panels for predicting concurrent disease severity (as measured by PUCAI or PGA), and for longitudinal disease tracking using PUCAI or the PGA index.

Although a couple of additional stool proteins, such as calprotectin, hemoglobin, haptoglobin, and properdin, also exhibit predictive potential for concurrent or future disease activity particularly in multi-marker panels, they do not match the predictive potential of fecal MMP-8, TIMP-2, fibrinogen, and PGRP-S, especially after correction for patient demographics and medication use. Although these findings need to be validated in additional patient cohorts, the stool proteins reported in this communication exhibit the potential for clinical use in several different ways. Fecal MMP-8, TIMP-2, PGRP-S, and Fibrinogen show a significant positive correlation with disease activity, as assessed by PUCAI or PGA scores, alluding to their potential utility in monitoring disease progression during follow up. Baseline levels of these stool proteins show significant ability to predict remission using previously described remission scales and are superior to fecal calprotectin in predicting these outcomes. Hence, they may be used to predict response to drug therapy, and this information could be used to reevaluate treatment options for patients unlikely to respond to standard of care treatment. Given their positive correlations with PGA endoscopy scores, these fecal proteins may potentially serve as pretests prior to endoscopy. A potential limitation of this study was not having more endoscopic severity data in the longitudinal cohort to define other clinical outcomes. Finally, some of these proteins, specifically fecal PGRP-S, may be useful for the screening of high-risk populations, given its superior sensitivity for CD and UC.

Several aspects of this study could be improved upon and expanded. Fecal MMP-8, PGRP-S, TIMP-2, and fibrinogen and multi-marker panels encompassing them need to be validated in additional cross-sectional and longitudinal cohorts of pediatric and adult UC patients, in order to confirm if they are indeed superior to fecal calprotectin. Eventually, randomized clinical trials using these biomarkers as indices to monitor treatment response are warranted. These proteomic observations need to be paired with gene expression studies from the same subjects in order to ascertain the likely origins of these elevated molecules. Given its genetic disease association in IBD, and its documented role in shaping the intestinal microbiome, fecal PGRP-S levels need to be examined in tandem with the genomic and microbiome profiles of these patients, in order to fathom the pathogenic relevance of PGRP-S in IBD. Finally, given that none of the validated markers in this study reliably distinguish UC from CD, the quest for such biomarkers should continue, taking advantage of the latest advances in OMICs technologies.

## Methods

### Human samples

Three cohorts of pediatric IBD were used in this study. The first 2 cohorts of patients were recruited from Children’s Healthcare of Atlanta/Emory University School of Medicine, Atlanta, GA. The first cohort of 24 subjects (10 CD, 5 UC, and 9 healthy controls (HC)) was used for initial IBD screening using the aptamer-based panel. The second is a cross-sectional cohort of 73 established IBD subjects (39 CD, 10 UC, and 24 HC; Supplementary Table 1). Written informed consent was obtained from the parents of all study participants, and this study was approved by the institutional review boards of Emory University School of Medicine and the University of Houston. Also, all study design and conduct complied with all relevant regulations for the use of human study participants and was conducted in accordance with the criteria set by the Declaration of Helsinki.

Further validation was performed using a prospective longitudinal cohort of 50 pediatric UC patients aged 4–17 years from the PROTECT study^44^. This multicenter inception cohort recruited 431 treatment naïve UC patients at 29 centers in the USA and Canada. This cohort was prospectively followed for a year where baseline (before treatment) and subsequent biosamples were obtained during the treatment period. Detailed patient selection criteria, study protocol, approvals, and clinical metrics assessed have been reported previously^44,45^, and summarized in Supplementary Table 2. Here, we selected a subset of 50 PROTECT participants where 4-time points stool samples were available, including baseline (week zero), week 4 (WK4), week 12 (WK12), and week 52 (WK52).

In PROTECT the diagnosis of UC for each participant was established using accepted clinical, endoscopic, and histological parameters^46^, disease extends beyond the rectum, a baseline Pediatric Ulcerative Colitis Activity Index (PUCAI) score of at least 10, with no previous therapy for colitis. PUCAI less than 10 denoted inactive disease or remission, 10–30 denoted mild disease, 35–60 denoted moderate disease, and 65 or higher denoted severe disease. Further, disease severity was defined using the PUCAI (range 0–85) and physician global assessment (PGA) grade ranging from 0–3 indices. PGA 0 denoted inactive disease or remission, 1 denoted mild disease, 2 denoted moderate disease, and 3 denoted severe disease. Depending on the initial PUCAI score, patients received initial treatment per protocol with either mesalamine (mild disease) or corticosteroids (moderate and severe disease). Escalation to immunomodulators (IM) or biologics was at the treating physician’s discretion. A detailed description of treatment guidelines is provided in Hyams et al.^44,45^. The PROTECT study was approved by the Institutional Review Boards at each of the participating PROTECT sites. All relevant ethical regulations for work on human participants have been met and conducted in accordance with the criteria set by the Declaration of Helsinki. Informed consent was obtained from the parents of all study participants. An overview of the study flow is depicted in Fig. 1.

### Stool extraction

Stool samples were weighed and added to an extraction buffer, vortexed for 1-minute alternating with a 5 min ice bath incubation until no fecal granules were visible. Following two rounds of centrifugation, the supernatant fraction was collected, assayed for protein content, and frozen in aliquots at −80 °C until the assay.

### Aptamer-based targeted proteomic screen of IBD stool

The stool extract was diluted to 20 μg/mL and subjected to the aptamer-based targeted proteomic screen using a library of 1129 validated aptamers (Somalogic Inc., Boulder, CO, USA), as detailed in our previous study^16^. Briefly, the sample was added to aptamer-coated beads allowing for the proteins in the sample to bind to their aptamer cognates. Next, the unbound proteins were washed away and the remaining bound proteins were biotinylated. The aptamer-protein complexes were photocleaved from the original beads and then conjugated to a second streptavidin-coated bead. The proteins were then denatured allowing for the recovered aptamer oligos to be hybridized onto a custom Agilent DNA array overnight, using Agilent buffers (Agilent 5188−5221) and scanned using a microarray scanner (Agilent G4900DA). Data were extracted using Agilent Feature extraction software. Along with the stool samples, eight controls were included to allow for quality control and normalization. A “no protein” buffer blank allowed for the assessment of the background signal.

### ELISA validation of stool protein biomarkers

In total, 33 proteins were initially selected from the aptamer-based screen for ELISA validation in a cross-sectional cohort. After initial testing for optimal sample dilution to use, 20 protein biomarkers were assayed using commercially available ELISA assays, following manufacturer instructions. Vendor, catalog number, and stool sample dilution for these ELISA kits are listed in Supplementary Table 3. The absolute levels of stool protein biomarkers were determined using standard curves run on each ELISA plate, and the data were normalized by stool weight. ELISA assay protocols are detailed in our previous studies^16^.

### Data analysis of the aptamer screening and ELISA results

Screening data were subjected to hybridization and median normalization, as detailed previously^16^. R Version 1.0.136 with the readxl, stats, and hmisc packages were used for further data analysis. All data were log-transformed. A nonparametric two-sided Mann−Whitney U-test was used to identify proteins that were significantly different between the subject groups. Statistical *p*-values and *q*-values (*p*-values adjusted for the false discovery rate, for multiple testing correction) were computed for each biomarker. Heatmaps were generated where hierarchical clustering using Euclidian distance was used for clustering of proteins. Ingenuity Pathway Analysis (IPA) was used to identify putative networks of interrelated proteins. For selecting proteins for ELISA validation, proteins were selected from each individual heatmap cluster and IPA network. Random Forest Classification analysis was performed using R to identify the relative importance of each biomarker candidate in disease classification, using the GINI index. The top 20 most discriminatory stool proteins with the largest impact on distinguishing IBD subjects from healthy controls were identified and ordered by their GINI coefficient.

### Statistical analysis of longitudinal data

Statistical analysis was performed on log_2_-transformed and standardized (centered at 0 with a variance of 1) values of all stool protein markers tested against the clinical activity and outcomes, across various time points. Pearson correlation analysis, the ANOVA test, the Mann-Whitney Wilcoxon test, and the Student’s *t*-test were performed using the respective R packages and the shown figures were generated using *ggcorrpolt, ggplot*, and *ggpubr* functions in R. To determine which stool biomarker best tracked with PUCAI or PGA disease activity, we first ran multilevel linear models with patient IDs as random intercepts regressing on each individual biomarker, using the lme4 and bbmle packages in R. Next, we performed elastic-net regularized regression, using glmnet package in R (version 3.6.2), adjusting for demographics (age, ethnicity, and gender) and medication use. Biomarker panels that best tracked with longitudinal disease activity scores were determined by Bayesian generalized multilevel models using brms package in R. Longitudinal biomarkers were entered as time-varying covariates and the models included random intercepts for subjects (to account for within-subject correlation). The horseshoe prior (df = 3, par_ratio = 0.5) was used to induce shrinkage, and gaussian and proportional odds models were used for PUCAI and PGA disease activity metrics, respectively. The performance of the different markers, panels, and models were compared using ROC AUC c-statistic and “Accuracy”, where Accuracy (prediction accuracy) was defined as (True positives + True negatives)/(Total number of participants) based on the classification table. Optimal cut-off values were derived using Youden’s index.

### Reporting summary

Further information on research design is available in the Nature Research Reporting Summary linked to this article.

## Supplementary information

Supplementary information

Reporting Summary

## Data Availability

The patient-level source data underlying Tables 1 and 2 and supplementary Tables 1 and 2 are available from the corresponding author upon reasonable request to maintain patient confidentiality. All other data supporting the findings of this study are available within the paper and its supplementary information files. Source data are provided with this paper.

## Source data

Source Data

## Code availability

Various R packages were used, and no unique code was written for this work.
