# Supplementary material for: Predicting disease course in ulcerative colitis using stool proteins identified through an aptamer-based screen
Source: Nat Commun. 2021 Jun 28;12:3989. doi: 10.1038/s41467-021-24235-0 (PMC8239008; doi:10.1038/s41467-021-24235-0)
Supplement: Supplementary file 1 — Supplementary information [file 41467_2021_24235_MOESM1_ESM.pdf]

Supplementary Table 1: Clinical and demographic information of 73 subjects, whose stool was used for Elisa validation

	Control (N=24)	Crohn's Disease (N=39)	Ulcerative Colitis (N=10)	Total (N=73)
**Gender**				
Female	12 (50.0%)	14 (35.9%)	7 (70.0%)	33 (45.2%)
Male	12 (50.0%)	25 (64.1%)	3 (30.0%)	40 (54.8%)
**Race**				
African American	12 (50.0%)	12 (30.8%)	3 (30.0%)	27 (37.0%)
Caucasian	11 (45.8%)	27 (69.2%)	3 (30.0%)	41 (56.2%)
Hispanic	0 (0.0%)	0 (0.0%)	2 (20.0%)	2 (2.7%)
other	1 (4.2%)	0 (0.0%)	2 (20.0%)	3 (4.1%)
**PUCAI/PCDAI**				
Mean (SD)	NA	11.92 (15.20)	41.50 (33.34)	12.05 (20.73)
Range	NA	0 - 45.0	0 – 85.0	0 - 85
**Disease Severity**				
Control	24 (100.0%)	0 (0.0%)	0 (0.0%)	24 (32.9%)
Mild	NA	7 (17.9%)	3 (30.0%)	10 (13.7%)
Moderate	NA	3 (7.7%)	0 (0.0%)	3 (4.1%)
Severe	NA	6 (15.4%)	5 (50.0%)	11 (15.1%)
Remission	NA	23 (59.0%)	2 (20.0%)	25 (34.2%)

Supplementary Table 2: Clinical and demographic information pertaining to 50 subjects drawn from the PROTECT cohort, whose stool samples were examined serially at four time-points (n=200 samples)

	**Baseline (N=49)**	**4wk FU (N=50)**	**12wk FU (N=50)**	**52wk FU (N=50)**	**Total (N=199)**
**Race**					
Black	2 (4.1%)	-	-	-	-
White	47 (95.9%)	-	-	-	-
**Female**					
Female	27 (55.1%)	-	-	-	-
Male	22 (44.9%)	-	-	-	-
**Age**					
Mean (SD)	12.429 (3.440)	-	-	-	-
Range	4.000 - 17.000	-	-	-	-
**CRP**					
Elevated	17 (47.2%)	3 (13.6%)	5 (20.8%)	6 (19.4%)	31 (27.4%)
Normal **(<1)**	19 (52.8%)	19 (86.4%)	19 (79.2%)	25 (80.6%)	82 (72.6%)
**Albumin**					
Reduced	23 (46.9%)	4 (10.3%)	3 (7.3%)	5 (11.1%)	35 (20.1%)
Normal **(>3.7)**	26 (53.1%)	35 (89.7%)	38 (92.7%)	40 (88.9%)	139 (79.9%)
**ESR**					
Elevated	24 (50.0%)	12 (34.3%)	7 (18.9%)	11 (26.8%)	54 (33.5%)
Normal **(<20)**	24 (50.0%)	23 (65.7%)	30 (81.1%)	30 (73.2%)	107 (66.5%)
**Hemoglobin**					
Reduced	28 (57.1%)	16 (42.1%)	17 (41.5%)	NA	61 (47.7%)
Normal **(>12)**	21 (42.9%)	22 (57.9%)	24 (58.5%)	NA	67 (52.3%)
**PGA**					
None	0 (0.0%)	29 (58.0%)	35 (70.0%)	34 (68.0%)	98 (49.2%)
Mild	13 (26.5%)	20 (40.0%)	13 (26.0%)	12 (24.0%)	58 (29.1%)

Moderate	23 (46.9%)	1 (2.0%)	2 (4.0%)	3 (6.0%)	29 (14.6%)
Severe	13 (26.5%)	0 (0.0%)	0 (0.0%)	1 (2.0%)	14 (7.0%)
**PUCAI**					
Mean (SD)	50.612 (19.702)	7.400 (10.654)	6.300 (11.553)	9.000 (15.714)	18.166 (23.726)
Range	10.000 - 85.000	0.000 - 55.000	0.000 - 50.000	0.000 - 80.000	0.000 - 85.000
**PUCAI Category**					
Inactive	0 (0.0%)	31 (62.0%)	36 (72.0%)	33 (66.0%)	100 (50.3%)
Mild	11 (22.4%)	17 (34.0%)	12 (24.0%)	14 (28.0%)	54 (27.1%)
Moderate	22 (44.9%)	2 (4.0%)	2 (4.0%)	2 (4.0%)	28 (14.1%)
Severe	16 (32.7%)	0 (0.0%)	0 (0.0%)	1 (2.0%)	17 (8.5%)
**Disease Location (PARIS classification)**					
Proctosigmoiditis (E1)	7 (14.3%)	NA	NA	NA	7 (14.3%)
Left-sided colitis (E2)	5 (10.2%)	NA	NA	NA	5 (10.2%)
Extensive (E3)	9 (18.4%)	NA	NA	NA	9 (18.4%)
Pancolitis (E4)	23 (46.9%)	NA	NA	NA	23 (46.9%)
Unasessable (E9)	5 (10.2%)	NA	NA	NA	5 (10.2%)
**REMISSION_WK**					
No Remission	0	21 (42.0%)	18 (36.0%)	26 (52.0%)	65 (43.3%)
Remission	0	29 (58.0%)	32 (64.0%)	24 (48.0%)	85 (56.7%)
**REM_CAL_WK**					
No Remission	0	40 (80.0%)	37 (74.0%)	35 (70.0%)	112 (74.7%)
Remission	0	10 (20.0%)	13 (26.0%)	15 (30.0%)	38 (25.3%)
**CSFREE REMISSION**					
CSFREE Remission	0	0	22 (44.0%)	23 (46.0%)	45 (45.0%)
NO Remission	0	0	28 (56.0%)	27 (54.0%)	55 (55.0%)

PGA: Physician's Global Assessment score; Clinical remission at weeks 4, 12, and 52 was defined by PUCAI score <10, with no prior rescue therapy or colectomy. Clinical remission with normal calprotectin (Remission_Calprotectin) at weeks 4, 12, and 52 was defined as follows. Clinical

remission at week 4 with fecal calprotectin < 250, and 14-day CS-free clinical remission at week 12 with fecal calprotectin < 250, and 28-day CS-free clinical remission at WK52 with fecal calprotectin < 250. Corticosteroid-free remission (CS-free Remission) at WK12 was defined as clinical remission and not on corticosteroid therapy for a minimum of 14 days prior to the WK12 assessment time.

Supplementary Table 3: ELISA validation of 33 stool proteins significantly elevated in IBD stool, on the aptamer-based screen

Protein	ELISA Manufacturer	Stool Dilution	Notes
Adiponectin	Raybiotech	1:2	Selected for further validation in cross sectional cohort
Alkaline Phosphatase	Raybiotech	1:10	Not selected for validation in cross sectional cohort
Calprotectin/S100A8/A9	R&D Systems	1:100	Selected for validation as gold-standard
D-Dimer	Raybiotech	N/A	Not selected for validation in cross sectional cohort
DAF/CD55	Raybiotech	N/A	Protein was not detectable by ELISA in preliminary dilution testing
DCSIGNR	Raybiotech	N/A	Protein was not detectable by ELISA in preliminary dilution testing
Desmoglein-2	Raybiotech	N/A	Protein was not detectable by ELISA in preliminary dilution testing
Elastase	Abcam	1:5	Selected for further validation in cross sectional cohort
Ferritin	Raybiotech	1:5	Selected for further validation in cross sectional cohort
Fibrinogen	Immunology Consultants Laboratory	1:5	Selected for further validation in cross sectional cohort
GRN	Raybiotech	N/A	Protein was not detectable by ELISA in preliminary dilution testing
Haptoglobin	R&D Systems	1:50	Selected for further validation in cross sectional cohort
Hemoglobin	Raybiotech	1:5	Selected for further validation in cross sectional cohort
HSP 70	R&D Systems	N/A	Protein was not detectable by ELISA in preliminary dilution testing
IGFII receptor	R&D Systems	N/A	Protein was not detectable by ELISA in preliminary dilution testing
IGFIsR	R&D Systems	N/A	Protein was not detectable by ELISA in preliminary dilution testing
LG3BP	R&D Systems	1:15	Selected for further validation in cross sectional cohort
Lipocalin-2	Raybiotech	1:100	Selected for further validation in cross sectional cohort
Lysozyme	Abcam	1:10	Selected for further validation in cross sectional cohort
MMP-1	R&D Systems	N/A	Protein was not detectable by ELISA in preliminary dilution testing
MMP-12	Raybiotech	1:10	Selected for further validation in cross sectional cohort
MMP-8	R&D Systems	1:1000	Selected for further validation in cross sectional cohort

MMP-9	Raybiotech	1:100	Selected for further validation in cross sectional cohort
Myeloperoxidase	R&D Systems	1:4000	Selected for further validation in cross sectional cohort
PGRP-S	R&D Systems	1:100	Selected for further validation in cross sectional cohort
Properdin	Raybiotech	1:10	Selected for further validation in cross sectional cohort
Proteinase-3	R&D Systems	1:25000	Selected for further validation in cross sectional cohort
Resistin	R&D Systems	1:5	Selected for further validation in cross sectional cohort
SAP	Abcam	1:2	Not selected for validation in cross sectional cohort
SerpinA4/Kallistatin	R&D Systems	1:50	Selected for further validation in cross sectional cohort
TIMP-1	Raybiotech	1:10	Selected for further validation in cross sectional cohort
TIMP-2	Raybiotech	1:2	Selected for further validation in cross sectional cohort
TNFsRII	R&D Systems	N/A	Protein was not detectable by ELISA in preliminary dilution testing

Rationale for selection of proteins for ELISA validation: *The aptamer-based screen identified 48 proteins to be significantly elevated in the stool of both CD and UC subjects versus healthy controls; from this list of 48 proteins, 18 were chosen for ELISA validation, selected to represent proteins from all IPA networks and heat-map clusters identified. Two proteins were elevated in the stool of CD patients but not UC when compared to healthy controls and one of these (LG3BP) was also included for ELISA validation. 20 proteins were elevated in the stool of UC patients when compared to healthy controls while not being elevated in CD versus healthy controls; from this list of 20, 8 were chosen for ELISA validation including DAF/CD55, Hsp70, IGFβ receptor, IGFIsR, MMP-1, Properdin, TIMP-2, and TNFsRII. From the Random Forest analysis results, an additional four proteins, Adiponectin, DC-SIGNR, GRN, MMP-12 (Figure 2D) were selected for ELISA validation.*

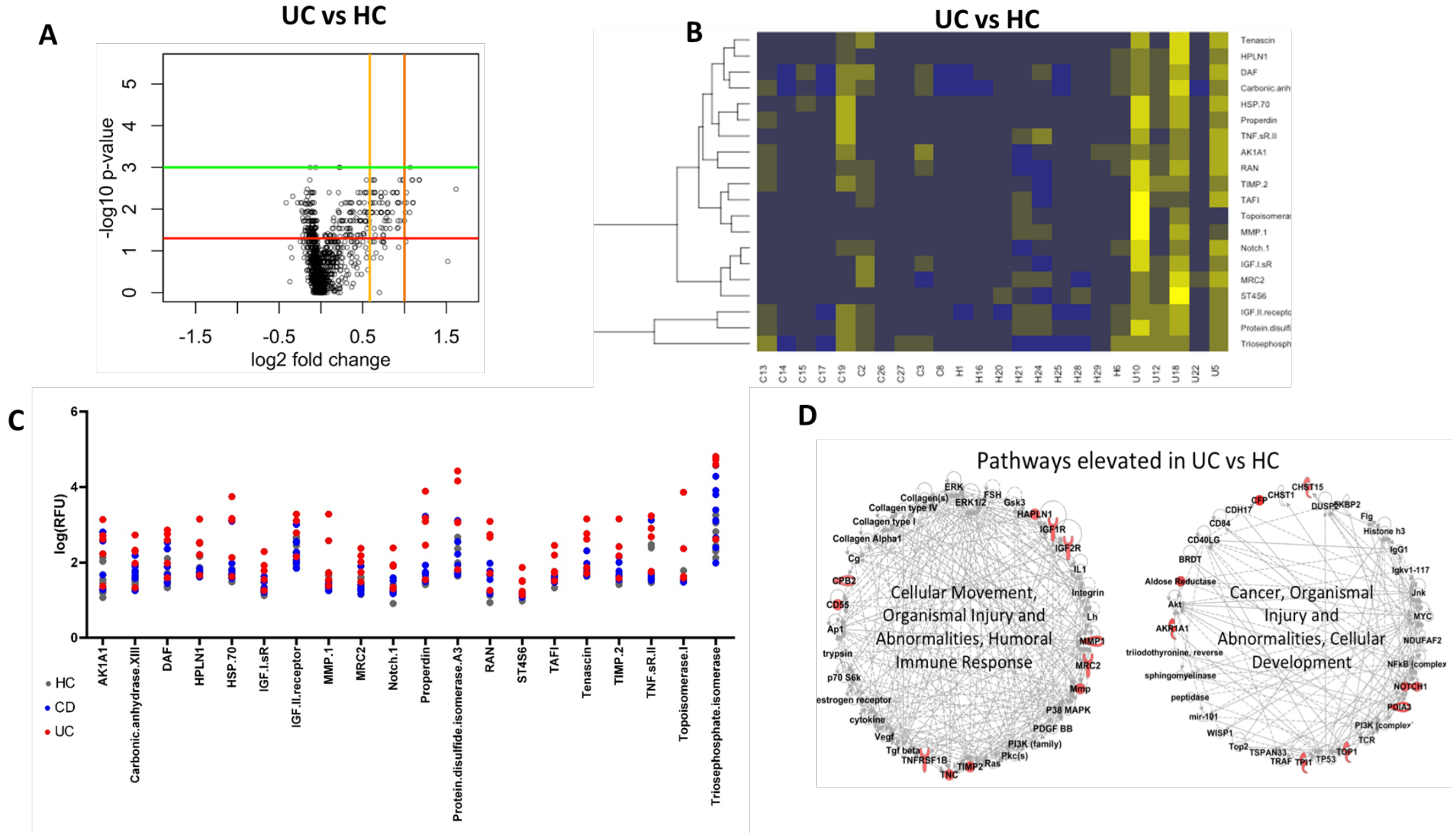

Supplementary Figure 1: Diagnostic markers of Ulcerative colitis: A) Volcano plot representation of results of the aptamer screen of 1129 human proteins revealed 120 proteins to be elevated in the stool of ulcerative colitis patients with FC >2, P <0.05 when compared to healthy controls. Thresholds for fold change are indicated with yellow and orange vertical lines for fold change > 1.5 and fold change > 2 respectively while thresholds for P value are indicated with horizontal red and green lines for P < 0.05 and P < 0.01 respectively.

Of these 120 proteins elevated in the stool of ulcerative colitis patients when compared to healthy controls (FC > 2, P <0.05), 20 proteins were found to be also elevated in ulcerative colitis patients when compared to Crohn's disease while not being elevated in Crohn's disease stool when compared to healthy controls as shown in Figure S1A. **B)**: A heatmap representation of the results of the aptamer-based screening showing the 20 proteins (P < 0.05, fold change >2) elevated in UC stool when compared to healthy control stool. The map shows the relative concentrations of these proteins in IBD patients and healthy controls. Each column represents a patient sample, while the rows correspond to the protein level measured using the aptamer-based screening assay. Proteins that are above the mean value (for each biomarker) are yellow, while those below the mean are blue, with proteins comparable to the mean value are shown in black. **C)**: The top 20 proteins (ranked in alphabetical order for UC versus healthy controls) that were significantly elevated in UC stool when compared to healthy control stool are shown in the dot plot where CD, UC, and healthy control subjects are shown with blue, yellow, and grey dots, respectively. **D):** Integrated Pathway Analysis display proteins that were significantly elevated in the stool of UC patients when compared to healthy control stool, and the functional pathways they belong to. The top 2 pathways were (1) cellular movement, organismal injury and abnormalities, humoral immune response and (2) cancer, organismal injury and abnormalities, cellular development. Proteins elevated in UC stool when compared to healthy control stool are shaded red. Documented and putative interactions between the displayed molecules are indicated by solid and dashed arrows, respectively.

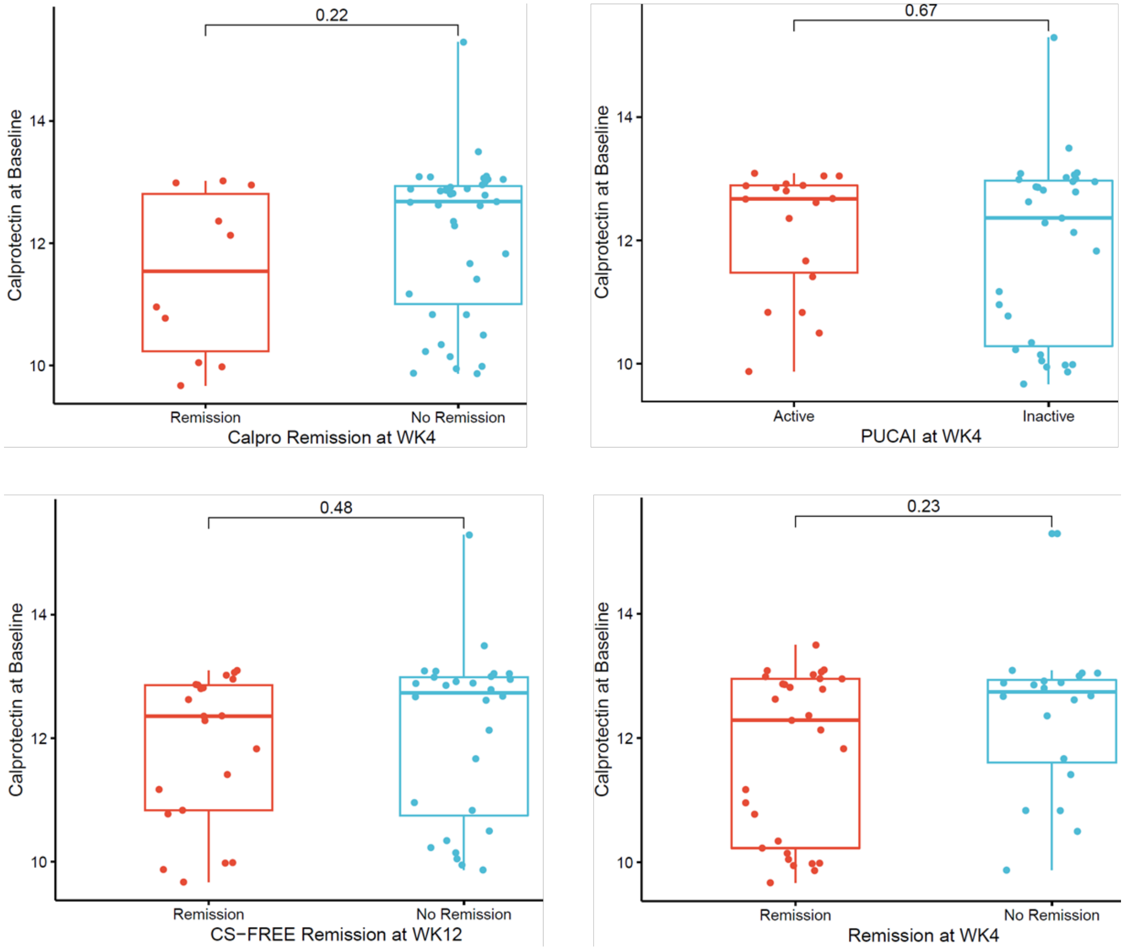

Supplementary Figure 2: All the significant clinical outcomes that were predicted by other four baseline stool markers were tested for Calprotectin. The X-axis shows the various clinical outcomes at WK4 and W12, and the Y-axis shows the log transformed protein expression values of calprotectin. None of the clinical outcomes were predicted by baseline calprotectin. Wilcoxon test was performed in each comparison.

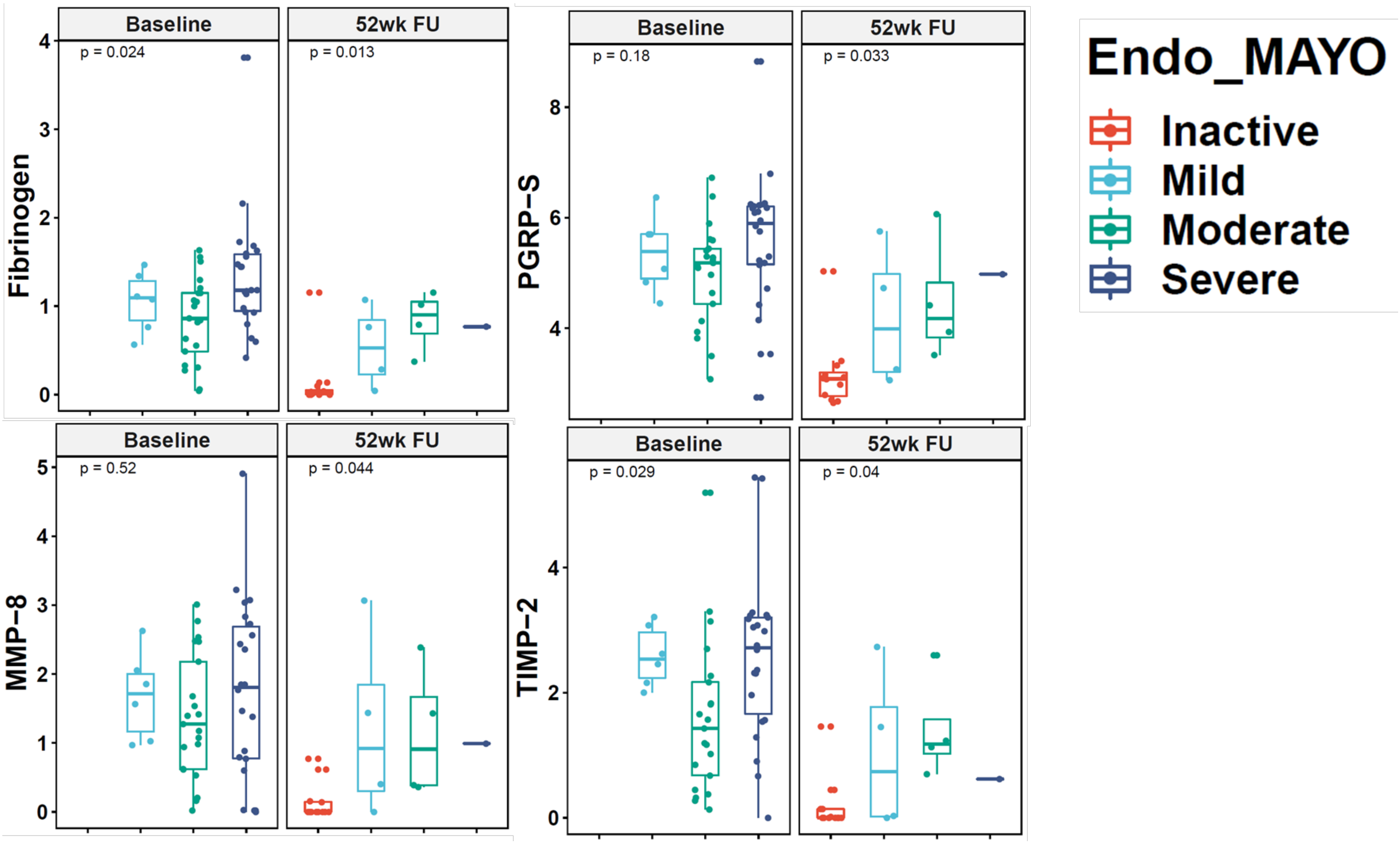

Supplementary Figure 3: All selected four baseline stool markers were tested for association of Endoscopic MAYO score at Baseline (n=50) and WK52 (n=21). The X-axis shows the disease activity group by endoscopic MAYO score, and the Y-axis shows the log transformed protein expression values of each marker. In each time point, the significant associations were tested for additive model from Inactive to Severe using ANOVA test.
