# Supplementary material for: Predicting disease course in ulcerative colitis using stool proteins identified through an aptamer-based screen
Source: Nat Commun. 2021 Jun 28;12:3989. doi: 10.1038/s41467-021-24235-0 (PMC8239008; doi:10.1038/s41467-021-24235-0)
Supplement: Supplementary file 2 — Reporting Summary [file 41467_2021_24235_MOESM2_ESM.pdf]

nature research

Corresponding author(s): DBPR NCOMMS-20-39207B

Last updated by author(s): Apr 8, 2021

Reporting Summary

Nature Research wishes to improve the reproducibility of the work that we publish. This form provides structure for consistency and transparency in reporting. For further information on Nature Research policies, see Authors & Referees and the Editorial Policy Checklist .

Statistics

For all statistical analyses, confirm that the following items are present in the figure legend, table legend, main text, or Methods section.

n/a	Confirmed	
☐	☒	The exact sample size (n) for each experimental group/condition, given as a discrete number and unit of measurement
☐	☒	A statement on whether measurements were taken from distinct samples or whether the same sample was measured repeatedly
☐	☒	The statistical test(s) used AND whether they are one- or two-sided<br>*Only common tests should be described solely by name; describe more complex techniques in the Methods section.*
☐	☒	A description of all covariates tested
☐	☒	A description of any assumptions or corrections, such as tests of normality and adjustment for multiple comparisons
☐	☒	A full description of the statistical parameters including central tendency (e.g. means) or other basic estimates (e.g. regression coefficient) AND variation (e.g. standard deviation) or associated estimates of uncertainty (e.g. confidence intervals)
☐	☒	For null hypothesis testing, the test statistic (e.g. F, t, r) with confidence intervals, effect sizes, degrees of freedom and P value noted<br>*Give P values as exact values whenever suitable.*
☐	☒	For Bayesian analysis, information on the choice of priors and Markov chain Monte Carlo settings
☐	☒	For hierarchical and complex designs, identification of the appropriate level for tests and full reporting of outcomes
☐	☒	Estimates of effect sizes (e.g. Cohen's d, Pearson's r), indicating how they were calculated

Our web collection on statistics for biologists contains articles on many of the points above.

Software and code

Policy information about availability of computer code

Data collection	No software was used to collect the data.
Data analysis	Biomarker data was plotted and analyzed using either GraphPad Prism 5 (GraphPad, San Diego, CA), Microsoft Excel 2016 (Microsoft Corporation), Integrated Pathway Analysis (IPA), or R 3.6.2 (R Foundation for Statistical Computing, Vienna, Austria).

For manuscripts utilizing custom algorithms or software that are central to the research but not yet described in published literature, software must be made available to editors/reviewers. We strongly encourage code deposition in a community repository (e.g. GitHub). See the Nature Research guidelines for submitting code & software for further information.

Data

Policy information about availability of data

All manuscripts must include a data availability statement. This statement should provide the following information, where applicable:

- Accession codes, unique identifiers, or web links for publicly available datasets
- A list of figures that have associated raw data
- A description of any restrictions on data availability

Source data are provided as Source Data files.

Field-specific reporting

Please select the one below that is the best fit for your research. If you are not sure, read the appropriate sections before making your selection.

☒ Life sciences ☐ Behavioural & social sciences ☐ Ecological, evolutionary & environmental sciences

Life sciences study design

All studies must disclose on these points even when the disclosure is negative.

Sample size	power calculations were used to predetermine the sample size
Data exclusions	No data were excluded from analyses.
Replication	All attempts of replication were successful.
Randomization	Samples were allocated into each group according to the clinical disease status.
Blinding	Blinding was not relevant. Clinical disease status was clear for each sample.

Reporting for specific materials, systems and methods

We require information from authors about some types of materials, experimental systems and methods used in many studies. Here, indicate whether each material, system or method listed is relevant to your study. If you are not sure if a list item applies to your research, read the appropriate section before selecting a response.

Materials & experimental systems

n/a	Involved in the study	
x		Antibodies
x		Eukaryotic cell lines
x		Palaeontology
x		Animals and other organisms
	x	Human research participants
x		Clinical data

Methods

n/a	Involved in the study	
x		ChIP-seq
x		Flow cytometry
x		MRI-based neuroimaging

Human research participants

Policy information about studies involving human research participants

Population characteristics	The first cohort of 24 subjects (10 CD, 5 UC and 9 healthy controls (HC)) were used for initial IBD screening with our aptamer panel. The second is a cross-sectional cohort of 73 established IBD subjects (39 CD, 10 UC, 24 HC; Supplementary Table S1). Further validation was performed using a prospective longitudinal cohort of 50 pediatric UC patients aged 4–17 years from the PROTECT study (Supplementary Table S2).
Recruitment	Three cohorts of pediatric IBD were used in this study. The first 2 cohorts of patients were recruited from Children's Healthcare of Atlanta / Emory University School of Medicine, Atlanta, GA. Further validation was performed using a prospective longitudinal cohort of 50 pediatric UC patients from the PROTECT study.
Ethics oversight	All patients gave informed consent, and this study was approved by the institutional review boards of Emory University School of Medicine and the University of Houston.

Note that full information on the approval of the study protocol must also be provided in the manuscript.
